# Across the consciousness continuum—from unresponsive wakefulness to sleep

**DOI:** 10.3389/fnhum.2015.00105

**Published:** 2015-03-10

**Authors:** Christine Blume, Renata del Giudice, Malgorzata Wislowska, Julia Lechinger, Manuel Schabus

**Affiliations:** ^1^Laboratory for Sleep, Cognition and Consciousness Research, Department of Psychology, University of SalzburgSalzburg, Austria; ^2^Centre for Cognitive Neuroscience Salzburg (CCNS), University of SalzburgSalzburg, Austria

**Keywords:** disorders of consciousness (DOC), electroencephalography (EEG), resting state, sleep, circadian rhythms

## Abstract

Advances in the development of new paradigms as well as in neuroimaging techniques nowadays enable us to make inferences about the level of consciousness patients with disorders of consciousness (DOC) retain. They, moreover, allow to predict their probable development. Today, we know that certain brain responses (e.g., event-related potentials or oscillatory changes) to stimulation, circadian rhythmicity, the presence or absence of sleep patterns as well as measures of resting state brain activity can serve the diagnostic and prognostic evaluation process. Still, the paradigms we are using nowadays do not allow to disentangle VS/UWS and minimally conscious state (MCS) patients with the desired reliability and validity. Furthermore, even rather well-established methods have, unfortunately, not found their way into clinical routine yet. We here review current literature as well as recent findings from our group and discuss how neuroimaging methods (fMRI, PET) and particularly electroencephalography (EEG) can be used to investigate cognition in DOC or even to assess the degree of residual awareness. We, moreover, propose that circadian rhythmicity and sleep in brain-injured patients are promising fields of research in this context.

## Introduction

In recent years, we have seen a rising number of patients who survive even severe brain injuries due to advances in intensive medical care. This process has been paralleled by a rising number of patients in altered states of consciousness. Usually, patients first enter a comatose state following severe brain injury, which is an acute and transitory state that only lasts for a limited amount of time. While some patients emerging from coma do immediately regain full consciousness, others progress through several states characterized by different levels of consciousness, the so-called disorders of consciousness (DOC). The amount of time that is spent in each of these states as well as the final outcome strongly varies across patients and etiologies. Traumatic incidents are usually related to a better prognosis with regard to recovery of awareness than non-traumatic (e.g., anoxic or ischemic damage) injuries (Working Party of the Royal College of Physicians, 2003).

**Figure 1:**
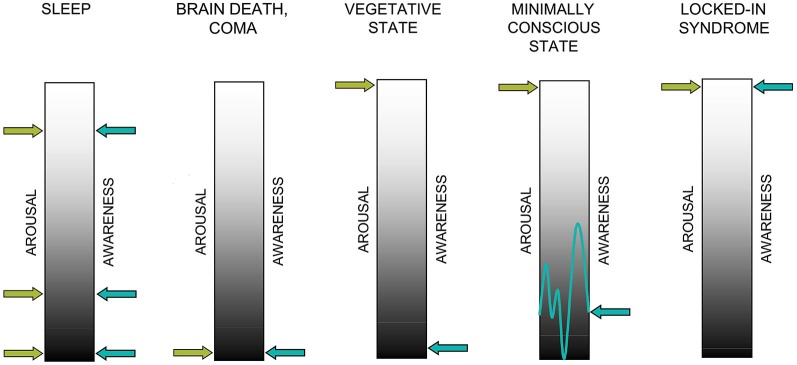
**Two factors contributing to consciousness: arousal and awareness**. In healthy sleep, arousal and awareness covary with the exception of rapid-eye movement (REM) sleep while in brain death and coma, arousal and awareness are not detectable. In the vegetative state (VS) arousal is preserved in the absence of evidence for awareness. In the minimally conscious state (MCS), both dimensions are present and behavioral evidence of awareness is reproducible albeit inconsistent. In the locked-in syndrome (LIS), both dimensions are more or less fully preserved despite complete loss motor functions (adapted with permission from Giacino et al. (2009)).

Commonly, consciousness is thought to require awareness of the self and the environment (i.e., contents of consciousness) as well as arousal at brain level. Both factors, awareness and arousal, are thought to be necessary for a conscious experience to emerge and neither of them is sufficient on its own (cf. Figure 1). This conceptualization forms the basis for behavioral as well as neuroscientific approaches to “assessing” consciousness in patients with severe brain injuries. In healthy individuals, arousal and awareness covary (with the exception of rapid eye movement (REM) sleep) whereas DOC states are characterized by a dissociation of the two: patients do recover periods of wakefulness following coma whereas awareness remains absent (VS/UWS) or impaired (MCS).

The question whether awareness or consciousness is an all-or-none phenomenon or rather a continuum is a matter of ongoing debate (see e.g., Overgaard et al., 2006; Overgaard and Overgaard, 2010; Fingelkurts et al., 2014). Studies using subjective introspection, that is studies asking participants to indicate whether they consciously perceived a stimulus or not, rather support the former view. However, neuroscientific studies suggest that when looking at brain responses, awareness can in fact be conceptualized as a continuum with different “levels of awareness” that have been suggested to correspond to a hierarchy of representational levels (Grill-Spector et al., 2000; Bar et al., 2001; Kouider et al., 2010). This idea is well in line with the notion that this hierarchy is reflected by the extent of activation in the neuronal network or “workspace” (Dehaene et al., 2006; Del Cul et al., 2007). Importantly, these studies also propose that there is a threshold, which has to be crossed for an experience to become accessible by introspection (Sergent and Dehaene, 2004a,b; Del Cul et al., 2007). Below this threshold, an experience may be subliminal or preconscious. However, although these experiences cannot be reported (verbally), we should not conclude that they do not contribute to a conscious experience, wherefore they may be considered “lower levels of consciousness” (Dehaene et al., 2006; Kouider et al., 2010). As this review focuses on the neuronal processes underlying awareness, we hereafter understand both dimensions arousal and awareness as a continuum.

The transition from coma to vegetative state (VS) is accompanied by a recovery of autonomic functions and the reemergence of arousal patterns or so called “sleep-wake cycles”. Although patients do have periods of eye-opening, they are by definition unaware of themselves and/or the environment and show no purposeful interaction with their environment (Giacino et al., 2009). Because arousal levels fluctuate and patients suffer from perceptual, attentional and motor deficits, the detection of signs of awareness is extremely challenging in this population.

The term “vegetative state” was originally chosen by Jennett and Plum (1972) to indicate that these patients have preserved vegetative nervous system functioning. To emphasize that these patients should be fully regarded as human beings and not “vegetating vegetables”, a new name for this neurological condition was recently presented by Laureys et al. (2010): the unresponsive wakefulness syndrome (UWS). This name, which is rather neutral and descriptive, refers to a clinical syndrome, which can be transitory or irreversible and includes patients who are not able to show voluntary and targeted motor behavior in the presence of eye-opening and sleep-wake cycles. In 2002, a new group of DOC patients was introduced to denote a group of patients who are neither continuously consciously awake nor in VS/UWS (Giacino et al., 2002). In contrast to VS/UWS, patients in the so-called minimally conscious state (MCS) do display inconsistent, but reproducible and discernible signs of awareness (i.e., command-following, yes-no responses and intelligible verbal expression). By definition, these patients are characterized by wakefulness and cyclic arousal as well as awareness of the self and/or the environment. Patients emerging from MCS can communicate reliably or use objects in a functional way. To do justice to the high interindividual variability among the group of MCS patients, it recently has been proposed to further subcategorize this clinical population in MCS minus (MCS−) and MCS plus (MCS+). Here, MCS− patients show e.g., sustained visual pursuit, localization of noxious stimulation or affective behavior contingent to relevant stimuli, whereas MCS+ patients show inconsistent but discernible command–following and thus evidence for “higher levels” of awareness (Bruno et al., 2011).

In a nutshell, coma and VS/UWS are both considered “unconscious” states as determined by conventional clinical assessment. They are—by definition—unresponsive to environmental stimulation (except for reflexive behavior) and fail to show purposeful behavior. MCS patients on the other hand show reproducible, but inconsistent signs of awareness of the self or environment (Giacino et al., 2002).

Patients in the locked-in syndrome (LIS) form yet another group that is behaviorally unresponsive to external stimulation. These patients are also unable to speak or move extremities, but in contrast to VS/UWS and MCS they are considered fully conscious and have more or less completely preserved cognitive functions. This condition often arises from neurological injuries or illnesses such as amyotrophic lateral sclerosis (ALS) that selectively disrupt motor pathways or reduce motor neuron functioning, respectively. The LIS is not a disorder of consciousness in the strictest sense (because awareness and arousal are retained), but can be mistaken for VS/UWS because of the behavioral similarities.

Until today, the gold standard for the clinical assessment of DOC patients remains the use of standardized behavioral methods such as the Glasgow Coma Scale (GCS; Teasdale and Jennett, 1974) or Coma Recovery Scale-Revised (CRS-R; Kalmar and Giacino, 2005). The crux of these methods, however, is, that they inherently rely on the patients’ ability to demonstrate their awareness to the examiner. From the absence of this ability it is concluded that a patient is not conscious. As severely brain injured patients are often not able to perform voluntary movements and motor responses to command (e.g., because of quadriplegia), the more or less exclusive use of these behavioral measures gives rise to an unacceptably high rate of misdiagnoses of up to 43% (Andrews et al., 1996; Schnakers et al., 2008b; Giacino et al., 2009). During the last decade, researchers have, therefore, tried to complement these classical methods with neuroscientific paradigms. With these paradigms, they hoped to find brain-based evidence of consciousness, i.e., evidence, which does circumvent the drawbacks of behavioral methods. Still, until today these new approaches have not been integrated in the conventional clinical assessment of DOC patients. Yet, interestingly science demonstrated that methods such as functional magnetic resonance imaging (fMRI), allow—in some cases—to assess the degree to which cerebral activity differs from healthy participants more objectively or even allow estimating the extent to which (higher) cognitive functions and even instruction-following is preserved in the absence of overt behavior (Monti et al., 2010). Bedside electroencephalography (EEG) is another assessment tool, which could be introduced more easily into clinical routine, is less stressful for patients and less costly. Current experiments using event-related potentials (ERPs) or time-frequency analyses as well as advanced methods such as EEG complexity measures investigate indicators of cognitive processing that may provide more precise information about the patient’s level of consciousness. The influence of neuroscientific advances becomes increasingly evident in the study of consciousness. We will, therefore, review current literature as well as recent findings from our group and discuss how classical neuroimaging methods (fMRI, positron emission tomography (PET)) and in particular EEG can shed light on preserved cognition and consciousness in DOC patients. Furthermore, we will review literature on sleep and circadian rhythmicity in severely brain injured patients and suggest new intervention methods to support rehabilitation as well as clinical assessment of awareness.

## Resting-state activity and consciousness

Investigation of resting-state brain activity in the absence of task instructions has been used in the hope of finding correlates of consciousness especially in those patients who lack the cognitive abilities (e.g., keeping information in working memory, comprehending language, being able to “execute” an action in some way) necessary for putting task instructions into action. The underlying idea is that indicators independent from the patient’s ability to follow instructions can also be used to assess the level of consciousness and make predictions about future developments.

The first studies of this kind mainly employed PET or fMRI. They were able to show for instance that unresponsive patients exhibit a global reduction of metabolism by 40–50% in VS/UWS compared to the range of values in healthy individuals and slightly higher, but comparable, in MCS (Laureys et al., 2004). Permanent VS (PVS; the term denotes patients who have been in VS for three or twelve months following non-traumatic or traumatic brain injury, respectively; Multi-Society Task Force on PVS, 1994), which is characterized by progressive trans-synaptic and Wallerian neuronal degeneration, was associated with even lower values of global brain metabolism (Laureys et al., 2004).

Another PET study revealed that VS/UWS patients show impairments in a network encompassing midline and associative regions (Laureys et al., 1999). Interestingly, the restoration of connectivity in PVS patients within exactly those regions (thalamic and associative regions) has been associated with later recovery of consciousness (Laureys et al., 2000). A study by Vanhaudenhuyse et al. (2011), moreover, suggests the presence of two distinct neuronal systems involved in mediating external (environmental) and internal (self-related) awareness. More precisely, two different systems have been identified: an extrinsic and an intrinsic system. The extrinsic system includes the lateral parietal and dorsolateral prefrontal cortices and is mainly involved in mediating external awareness (i.e., consciousness of external stimuli as well as awareness of the environment) while the intrinsic network comprises midline precuneus/posterior cingulate and mesiofrontal/anterior cingulate cortices and mediates internal awareness (i.e., stimulus-independent and self-related thoughts). Interestingly, other studies did not only confirm the existence of these two networks, but were also able to show that their integrity is related to varying levels of consciousness (Thibaut et al., 2012). In particular, VS/UWS patients showed dysfunctions in both the external and the internal network, while MCS patients showed impairments only in the internal network and thalamic regions when compared to healthy subjects.

Following these findings, other imaging studies further investigated resting-state networks with the aim of identifying specific consciousness networks. In this context, the default mode network (DMN), which includes the posterior cingulate and the medial prefrontal cortex as well as the posterior parietal cortices, gained particular attention. It has been linked to consciousness and awareness (Boly et al., 2008) in many studies and in particular to the processing of self-relevant information (for meta analysis see Qin and Northoff, 2011). The DMN seems to be functionally disconnected in brain death (Boly et al., 2009) and connectivity within the network has been suggested to decrease with the level of consciousness (Vanhaudenhuyse et al., 2010). Other studies validate the idea that functional connectivity between the areas constituting the DMN support consciousness by showing that connectivity of exactly those areas is drastically diminished in DOC (Cauda et al., 2009; Fernández-Espejo et al., 2012; Soddu et al., 2012). Another study using methods based on the functional architectonics theory of brain-mind functioning proposed by Fingelkurts et al. (2010), supports this view finding functional connectivity (termed “synchrony” in this theory) among neuronal modules including the DMN to be markedly decreased or even absent in VS/UWS and highest in conscious healthy individuals while patients in MCS exhibited intermediate synchrony (Fingelkurts et al., 2012). From a theoretical perspective, these findings are well in line with the global neuronal workspace model of consciousness (Dehaene and Naccache, 2001; Sergent and Dehaene, 2004b) as well as the information integration theory of consciousness (Tononi, 2004, 2008). The former theory proposes that the neural basis of consciousness is a “sudden self-amplifying process leading to a global brain-scale pattern of activity” that occurs once ignited, i.e., when a certain threshold has been crossed (Sergent and Dehaene, 2004b). The latter proposes that conscious experiences crucially depend on the brain’s ability to integrate information, which is reflected by functional connectivity among different brain modules. Interestingly, regions constituting the DMN have been found to be among the most well-connected regions in the brain (Cole et al., 2010). Besides this, the amount of DMN deactivation is inversely related to the probability of subsequent awakening from coma and has been found to correlate with the behaviorally assessed level of consciousness (Crone et al., 2011).

Until now it is evident that imaging studies of resting state data can provide insight into the level of consciousness in DOC patients. However, PET requires a special scanner only available in selected clinics as well as the injection of a radioactive glucose analogue. Moreover, although fMRI scanners are readily available nowadays, it is a costly method not all patients can be tested with (exclusion criteria are e.g., a cardiac pacemakers or metal pins and plates remaining in the patient’s body after surgical treatment). Different research groups thus investigated the suitability of the EEG for the exploration and interpretation of resting state activity. In one study, Lehembre et al. (2012) reordered 15 min of resting EEG in several DOC patients. They were able to show that resting state EEG of VS/UWS and MCS patients differs in the amount of delta and alpha power. VS/UWS patients were found to have more delta and less alpha power and, additionally, significantly decreased connectivity in the theta and alpha bands compared to MCS patients. A similar approach was used to determine whether different frequency bands and spectral analyses of the EEG can predict the behavioral outcome in DOC patients by Lechinger et al. (2013). This study demonstrated that the alpha/theta ratio as well as the resting EEG’s spectral peak was strongly correlated with the behavioral CRS-R score in DOC patients. The bispectral (BIS) index, a physiological index calculated from the weighted sum of several electroencephalographic subparameters (high-frequency (14–30 Hz) power, low-frequency synchronization and the presence of periods of nearly of completely suppressed EEG activity (i.e., isoelectric phases)) used to measure the depth of anesthesia, has also been used to differentiate between VS/UWS and MCS (Schnakers et al., 2008a). Additionally, the BIS during early stages of recovery has been related to good recovery one year later (Schnakers et al., 2005). Recently, also connectivity measures have been applied to resting state EEG data. Chennu et al. (2014) applied graph-theoretic measures to high-density EEG data from DOC patients and healthy controls. Looking at connectivity in canonical frequency bands, they found differences between patient networks and control participants. More precisely, patients’ alpha network modules were spatially limited and long-distance connections commonly observed in healthy networks were absent. Although these characteristics were reversed in the theta and delta bands, connectivity patterns were found to be robust across patients, which could be deemed the result of reorganization processes. Besides this, network metrics in the alpha band were found to correlate with patients’ behavioral CRS-R scores. Another class of measures that can be used to discriminate between different entities in DOC is entropy measures. The common idea behind EEG entropy measures is to describe the irregularity and complexity of an EEG signal and in the case of e.g., symbolic transfer entropy (STE) even to determine the direction of information flow. The underlying logic is that a regular EEG signal, such as the signal recorded in slow wave sleep or deep anesthesia, has a low level of entropy or complexity while the one recorded from awake subjects has a higher entropy level. Gosseries et al. (2011) were for example able to show that it is possible to define an entropy cut-off value that can be used to differentiate between unconscious states and MCS with a high level of specificity even though not being valid for prognostic purposes. Another interesting approach employs high-density EEG in combination with transcranial magnetic stimulation (TMS) to assess effective connectivity in DOC patients (Rosanova et al., 2012). This approach can be used to calculate the so-called perturbational complexity index (PCI; Casali et al., 2013), which provides a numerical quantification of the brain’s ability to support complex activity patterns. VS/UWS patients were found to show a simple localized response to the TMS stimulation pulse, which was reflected by low PCI values indicating low effective connectivity. In MCS, TMS was able to trigger more complex activations involving cortical areas distant from the site of stimulation, which was mirrored by intermediate PCI values. This suggests a more efficient interaction between different and distant brain regions, which is linked to a higher level of consciousness. Conscious wakefulness was associated with highest PCI values. The TMS-EEG approach is remarkably well-researched in healthy individuals across the consciousness continuum as well as in DOC patients (Casali et al., 2013; Sarasso et al., 2014). It moreover seems to be one of the very few measures that allow differentiating between different levels of consciousness on an individual level.

In summary, much effort has been undertaken in order to find reliable and valid measures for the assessment of residual resting state brain activity, which could serve as an indicator of awareness in DOC patients. Despite these advances, we still lack a clear and comprehensive understanding of human consciousness, which would allow for a better differentiation of DOC states. Moreover, most of the advances and developments of the last 20 years, have unfortunately not found their way into clinical routine. In the future, the development of new EEG paradigms should allow for a better differentiation of VS/UWS and MCS and enable a more reliable assessment and quantification of consciousness along its continuum. Finally, these efforts should flow into better rehabilitation guidelines in terms of appropriate treatments individually attuned to the patient’s needs and abilities. Moreover, developments in EEG methodology and analysis should aim at developing more sensitive and effective brain-computer interfaces (BCIs) allowing MCS patients with sufficient, but rather limited cognitive abilities to communicate with the outside world.

## Studying consciousness using passive stimulation and active electroencephalography paradigms

Besides resting-state brain activity in the absence of stimulation or task instructions, ERPs, that is, responses in the EEG evoked by and time-locked to the presentation of a stimulus, have been used to assess cognitive processing and predict outcome in DOC patients. They depend to a varying degree on the physical properties (e.g., pitch, volume or font size) of the stimuli presented as well as top-down cognitive processes such as selective attention (for a review see Herrmann and Knight, 2001). Early components of the ERP usually occur within the first 100 ms after stimulus onset and are known to persist even in unconscious states such as sleep or anesthesia. On the contrary, later components such as the P300 as well as indexes of verbal semantic processing (N400, late positive component (LPC)) have been proposed to be more strongly related to conscious information processing (Kotchoubey, 2005; Rohaut et al., 2015).

Among the earliest components of the ERP, somatosensory evoked potentials (SEPs) have been used to predict outcome in DOC. It was found that for example bilateral absence of SEPs elicited by median nerve stimulation within 8 days after traumatic brain injury (TBI), is predictive of a negative outcome, that is VS/UWS or death. Furthermore, it has been shown that abnormalities of brainstem auditory evoked potentials (BAEPs) or short-latency SEPs indicate a high probability of a negative outcome. For example, Amantini and colleagues were able to quantify the deterioration of brain functions in patients suffering from severe brain injuries using SEPs and also demonstrated a close relationship between SEPs and the evolution of the patients’ clinical state (Amantini et al., 1992). Besides these very early components, also later ERP components have been used to investigate cognitive processing and make predictions about recovery from coma. The presence of the mismatch negativity (MMN) for example has a strong positive prognostic value in severely brain injured patients (Naccache et al., 2005). The MMN is an ERP component, which can be elicited by the presentation of a deviant stimulus (e.g., a higher pitched tone) in a sequence of identical stimuli. In one study, Luauté et al. (2005) showed that when an MMN could be detected during early stages of coma, the estimated probability for a good functional outcome was around 70%. Also, the presence of a P300 has been linked to preserved attentional as well as working memory capacities and hence is frequently used in the assessment of DOC patients. The presence of both early and late components (e.g., N100 and P300) in DOC patients has also been linked to a good clinical outcome, although longer and more variable latencies as compared to healthy individuals suggest overall impaired information processing (e.g., Perrin et al., 2006). In a meta-analysis Daltrozzo et al. (2007) compared the prognostic value of several ERP components. They concluded that the presence of the N100, MMN and P300 components is a highly significant predictor of awakening with the later components (i.e., MMN and P300) having significantly more predictive power than the early N100. Another prognostic factor, which has to be taken into account, is the etiology of brain injury, where the probability of regaining awareness following VS is higher in traumatic and post-operative etiologies than in anoxia and metabolic pathologies (Working Party of the Royal College of Physicians, 2003). This is probably due to the former rather leading to localized injuries, which can often be compensated for by other brain regions, while the latter usually leads to widespread and more diffuse damage.

Besides a valid prognosis of a patient’s future development, it is also important to discriminate between different levels of consciousness and to further discern DOC patients on the basis of their residual cognitive functions. This may have important implications for rehabilitation efforts and sometimes even guide decisions about life or death of a patient. To this end, Fischer et al. (2010) investigated several ERP components in PVS and MCS patients. In MCS patients somatosensory N20-P24 components and the auditory N1 generally had a higher prevalence than in PVS patients, whereas the presence of later components was not statistically different between the two groups. In another study, Perrin et al. (2006) reported that in all LIS and MCS and 60% of the VS/UWS patients, the P300 was elicited by the passive (i.e., without further task instructions) auditory presentation of the patient’s first name (SON; subject’s own name). The P300 latency was significantly delayed in MCS and VS/UWS compared to LIS patients and controls. The authors conclude that, especially for salient stimuli such as the own first name, semantic processing is at least partially preserved in non-communicative brain-damaged patients, although this process seems to be delayed in MCS and VS/UWS. The electrophysiological responses in this study did, however, not allow for a discrimination between the two groups of DOC patients. More recently, several studies also investigated the diagnostic usefulness of indexes of semantic processing in severely brain-injured patients (see e.g., Kotchoubey et al., 2005; Schabus et al., 2011; Steppacher et al., 2013). Of special interest in these studies was the N400, an ERP indexing violations of semantic congruity (Kutas and Hillyard, 1980) in word pair paradigms. Since the N400 has been shown to occur even in the absence of consciousness (Sergent et al., 2005), a study by Rohaut et al. (2015) also investigated a later ERP, the late positive component (LPC, also termed P600), as an index of conscious semantic processing. However, although several studies found group differences between VS/UWS and MCS patients, on a single-subject level, neither N400 nor LPC do seem to be a valid index of the level of awareness for even in conscious controls they often cannot be observed (Rohaut et al., 2015).

Another approach to analyzing EEG data obtained during passive auditory stimulation are entropy measures, which have also been applied to resting state data (see section resting-state activity and consciousness). For example, King et al. (2013) investigated the amount of information shared between brain regions during passive auditory stimulation with tones as quantified by weighted symbolic mutual information (wSMI). They were able to show that wSMI is related to the level of consciousness in DOC being even able to differentiate MCS from VS/UWS. In our own research, we were able to show that, in a passive SON task, both permutation entropy (PE) as a measure of local information processing as well as STE as a measure of directional information transfer are suitable indicators for the level of consciousness in DOC (Lechinger et al., 2014).

Recently, it has been argued that passive paradigms (i.e., paradigms investigating patients’ brain activity at rest or during presentation of stimuli that do not require active collaboration) are not useful for differentiating between MCS and VS/UWS patients. This is because passive paradigms often use stimuli (i.e., SON or odd stimuli in a sequence of identical ones) that might well elicit automatic or even conditioned responses (i.e., automatic semantic processing or a conditioned orienting response to one’s own name). Thus, evoked brain responses in passive paradigms do not allow for unambiguous conclusions regarding the level of consciousness. To counteract this methodological issue and to better disentangle automatic or unconscious from voluntary or conscious brain activity, it has been proposed to always combine passive and active tasks (Schnakers et al., 2008b). In the latter task, patients are asked to actively follow an instruction, e.g., to count how often a stimulus is presented. If a patient successfully follows such an instruction, different cognitive functions such as sustained and selective attention and inhibition of interfering processes as well as working memory need to be involved. This suggests that if a patient is able to follow instructions, this is a very strong indicator of preserved higher cognitive functioning and thus conscious awareness. One way to assess instruction-following in non-communicative patients is by means of electrophysiological (EEG) responses. The P300 is for example very sensitive to top-down selective attention with its amplitude varying as a function of task instruction. Thus, it can be used to detect instruction-following and hence conscious processing in DOC patients. In a study by Schnakers et al. (2008b), patients in altered states of consciousness were instructed to count the SON or another target name or listen passively to the stimuli. A higher P300 amplitude was found in response to the to be counted target stimuli in MCS patients, but not in VS. Interestingly, this pattern was even found in MCS patients at the lower end of the MCS spectrum, i.e., patients only showing low behavioral responses such as visual fixation and pursuit. This suggests that the P300 in paradigms including passive and active conditions may support the detection of voluntary information processing in DOC. A more recent study, however, advises a cautious interpretation of these findings as volitional top-down attention can be impaired in patients with covert cognition (Schnakers et al., 2014). This questions the specificity of the task as it could lead to false negative results.

Following results of studies of attention in healthy individuals, other authors investigated two subcomponents of the P300, namely P3a and P3b. While the frontal novelty P3a is thought to index exogenous “bottom-up” attention, the posterior P3b is thought to reflect endogenous “top-down” attentional processes (e.g., Squires et al., 1975; Polich, 1988, 2007). Following this idea, the investigation of the two subcomponents could allow for a more exact evaluation of the patients’ cognitive abilities. A recent study by Chennu et al. (2013), however, does not fully support advantages of this differentiation. In a task, which asked patients to count the number of times a specific word (YES vs. NO) was presented, only 3/12 MCS and 1/9 VS patients showed a P3a to the target word. Although the same VS patient, who exhibited a P3a, also showed a P3b response suggesting volitional control and hence challenging the VS diagnosis, no single MCS patient exhibited a P3b. This was despite some of the MCS patients even showing command-following behavior on other tasks. In conclusion, these findings rather question the usefulness of the distinction between P3a and P3b for the assessment of cognitive processing in DOC patients.

**Figure 2:**
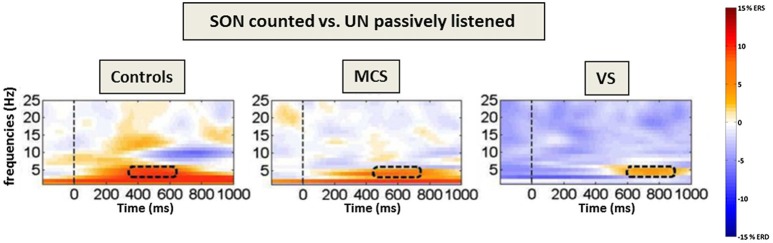
**Frontal theta event-related (de-)synchronization (ERD/ERS) to counted own names**. Time–frequency difference plots [targets (SON)-passively listened other names] for the “count own name”-condition. The dashed lines mark the presentation of the stimuli (names) and the rectangles the area with the highest difference in the theta-range. Note the increasing processing delay in theta power over groups as well as the alpha ERD in controls only (adapted with permission from Fellinger et al., 2011).

Fellinger et al. (2011) did not focus on ERPs, but investigated the oscillatory activity by means of time-frequency analyses in the lower frequency range (i.e., theta and alpha oscillations, which have been linked to attention and memory processes (Klimesch, 1999)). This method is thought to be advantageous for investigating brain processes in DOC patients as it does not require brain responses to be strictly time-locked to an event (cf. Figure 2).

Results indicate that MCS as well as VS/UWS patients respond with an increase in theta event related synchronization (ERS) when they are asked to focus and count the SON, but not when they are asked to listen passively to the stimuli. Thus, also time-frequency analyses allow for conclusions regarding the patients’ ability to follow instructions. Höller et al. (2011) moreover proposed the use of time-frequency analyses in combination with a single-subject approach for a more sensitive and reliable analysis of EEG responses in patients. More generally, time-frequency analyses allow to focus on distinct cognitive processes reflected by the behavior of oscillations in different frequency bands and to thereby refine the understanding of residual cognitive processing in DOC.

Recent studies have demonstrated that emotionally and self-relevant stimuli can better engage attentional resources and consequently also induce stronger “higher-order” neuronal responses in DOC patients (Jones et al., 1994; Di et al., 2007; Di Stefano et al., 2012). We propose that this could be beneficial for the detection of retained cognitive functioning in this patient population. In a study from our group (del Giudice et al., 2014a), we investigated whether the use of a familiar voice in the traditional own name paradigm boosts the engagement of attentional resources. Results from healthy controls indicate that the passive presentation of self-relevant stimuli (SON and stimuli uttered by familiar voices) induces stronger alpha ERD than the presentation of non-self-relevant ones. This suggests that self-relevant stimuli are able to attract attention and possibly also trigger processes involved in recognizing and accessing self-referential information. Furthermore, in the active condition we were able to differentiate between targets and non-targets on the basis of delta and theta ERS as well as alpha ERD irrespective of the familiarity of the voice. Thus, also in this version of the paradigm, time-frequency analyses allow to reliably assess instruction-following. Preliminary analyses of data from a VS patient revealed higher theta ERS for the familiar voice as well as for the SON (Schabus et al., 2014). These findings indicate that self-relevant information was able to attract the patient’s attention–at least on a bottom-up level. In the active condition, theta synchronization following stimulus presentation was evident and even specific for target stimuli.

In a similar study, we contrasted angry and neutral voices in order to understand whether emotional prosody is also able to engage attention and hence to facilitate top-down cognitive processes (del Giudice et al., 2014b). Preliminary results from a healthy sample indicated that in the passive condition, stronger alpha ERD to angry than to neutral voice was evident irrespective of the stimulus (SON vs. unfamiliar name) presented. This is well in line with the results obtained using familiar voices. Again, instruction-following gave rise to a differential EEG response pattern. Altogether, results from the two studies support the notion that adding emotional content to stimuli increases their salience and thus benefits the detection of residual cognitive functioning in DOC patients.

A recent study by Sitt et al. (2014) systematically compared 14 electroencephalography markers in a large cohort of DOC patients. They found that theta and alpha band power as well as measures of EEG complexity (permutation entropy and Kolmogorov-Chaitin complexity) and information exchange (phase locking index and wSMI) constitute the most reliable measures of the state of consciousness. The combination of several of these markers allowed for an even more accurate discrimination between consciousness levels.

A different class of active EEG paradigms makes use of the effects imagining motor actions has in the brain, which can be visualized using EEG and fMRI. Owen et al. (2006) were able to show in an fMRI study that even a patient behaviorally diagnosed with VS/UWS was able to imagine to “play tennis” or “navigate through a house” at will and that this method can even be used by patients to communicate with their environment in the fashion of yes/no answers (Monti et al., 2010). In the EEG domain, it has similarly been shown that some DOC patients are able to respond to motor imagery instructions such as to imagine swimming or walking around their home (Goldfine et al., 2011), to move hands and toes (Cruse et al., 2011) or to grasp a cup (Lechinger et al., 2013) with changes in EEG activity. Some of these findings have already been investigated regarding the suitability for BCIs (e.g., Monti et al., 2010). In a study from our group (Körner et al., 2014), we investigated EEG activation patterns in response to watching or imagining a fist or tongue movement in healthy individuals. Preliminary analyses revealed alpha and beta ERD above left sensorimotor areas during fist movement observation as well as weaker, yet observable alpha ERD during imagery. Observing and imagining tongue movements led to bilateral synchronization in the alpha band above the same areas. These findings underline the potential suitability of EEG-based motor imagery paradigms for bedside communication with patients. In summary, we can conclude that ERPs and oscillatory patterns, especially in combination with active paradigms, seem to be promising tools for disentangling voluntary or conscious from automatic or unconscious brain responses in DOC patients. They are thus helpful in assessing preserved brain functioning and in differentiating MCS and VS/UWS patients. These paradigms do, however, have drawbacks that decrease the reliability and validity of their results with regard to the assessment of consciousness. Although active paradigms do provide a promising approach for the detection of consciousness in brain-damaged patients, there may be patients, who are not able to follow instructions. This is because understanding and eventually executing an instruction is extremely demanding with regard to (higher) cognitive processes. Consequently, patients with compromised cognitive abilities may have moments of wakeful awareness, but are still not able to give “proof” of their awareness when tested with complex paradigms such as motor imagery.

## Circadian rhythmicity and sleep in disorders of consciousness

### Circadian rhythmicity

Another approach to investigating the integrity of brain functioning is to observe naturally occurring circadian rhythms. There are circadian variations of e.g., the level of arousal, temperature, blood pressure, hormone secretion, attention and cognitive abilities. These variations are endogenously controlled by a biological “Zeitgeber” located in the suprachiasmatic nucleus of the hypothalamus (SCN). In case the SCN is destroyed, circadian rhythmicity vanishes. In rats, for example, the total amount of sleep time remains stable, but length and timing of sleep episodes becomes instable (Ibuka and Kawamura, 1975). In DOC patients, reinstatement of circadian rhythmicity (i.e., sleep-wake cycles) is by definition characteristic for the emergence from coma to VS/UWS. Although re-emergence of circadian rhythms in DOC has generally been related to an improvement of the patient’s state, studies suggest that some rhythms recover while others do not. Studies have for example reported significant circadian changes in body temperature as well as urinary excretion of hormones and sodium in (P)VS/UWS patients, but no changes in blood pressure or pulse rate were observed (Fukudome et al., 1996; Pattoneri et al., 2005). A more recent study by Bekinschtein et al. (2009) emphasizes that the etiology of the brain injury has to be taken into account when investigating circadian rhythms. They found that VS/UWS patients with TBI, but not with anoxic-hypoxic origin exhibited circadian cycling of body temperature.

Besides these parameters, also arousal levels vary across the 24 hours of the day. A study by De Weer et al. (2011) investigated circadian arousal rhythms monitoring changes in the frequency and amplitude of hand movements by means of videography and wrist-actigraphy. They found circadian rhythmicity of arousal to be preserved in two MCS patients with TBI, but not in an MCS patient who suffered from anoxic-ischaemic brain damage and neither in a comatose patient. A recent investigation by Cruse et al. (2013) recorded the motor activity of a large sample of 55 DOC patients (18 VS/UWS and 37 MCS) and found significant circadian changes of motor behavior in 83% and 84% of VS/UWS and MCS patients, respectively. Yet, the authors stated that VS/UWS patients exhibited significantly less pronounced circadian rhythmicity than MCS patients.

Generally, these findings demonstrate the need to further investigate the presence of circadian rhythmicity in DOC patients, not only to gain insight into brain functioning, but also to validate the amount of circadian rhythmicity as an indicator of the level of consciousness. Future studies should also investigate changes in plasma hormone levels (e.g., melatonin, cortisol) and temperature and also include the assessment of blood pressure and heart-rate (variability) as indicators of circadian variations in DOC. Moreover, interventions aiming at reinstating circadian rhythmicity may be a promising tool to support assessment as well as rehabilitation in DOC patients.

### Sleep

The most readily observable circadian rhythm, which all mammals show, is the sleep-wake cycle. In DOC and here especially for the purpose of differentiating VS/UWS from coma, its presence is often assessed on the basis of prolonged periods of eye opening and closing. However, conclusions regarding circadian rhythmicity drawn exclusively from these behavioral observations or actigraphy might be misleading and often little reliable. We therefore propose to use polysomnography (PSG), a method combining EEG as well as eye movement, muscle activity and respiration measurements, for a reliable and valid assessment of sleep in patients. PSG recorded over 12 or 24 hours provides insight into the rhythmicity of sleep/wake patterns as well as sleep abnormalities. Moreover, PSG allows for a more fine-grained analysis of different sleep stages, and their characteristics, especially in clinical populations, and thus provides additional measures that might be helpful in characterizing a patient’s state and predicting the outcome (Van de Water et al., 2011).

**Figure 3:**
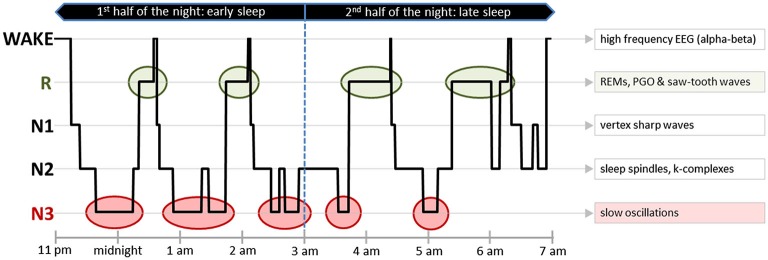
**Sleep pattern from a healthy human**. An exemplary hypnogram depicting the different sleep stages over 8 h of nocturnal sleep. On the right side of the figure, typical EEG graphoelements of every sleep stage have been listed. In addition to certain EEG patterns, some of the sleep stages also have characteristic EMG and EOG activity patterns. Early sleep is predominated by N3, whereas later sleep is characterized by a relatively high amount of R. One observes during wake—high muscle tone, high frequency EEG (alpha-beta) and blinks; during light N1—eye rolling and vertex sharp waves; during light N2—sleep spindles and k-complexes; during deep N3—slow oscillations; and during R—rapid eye movements, PGO waves, saw-tooth waves and muscle atonia with concurring rare muscle twitches. Abbreviations: EEG, Electroencephalography; EMG, Electromyography; EOG, Electrooculography; R, Rapid Eye Movement Sleep; REMs—Rapid Eye Movements; PGO, Ponto-Geniculo-Occipital Waves.

While healthy human sleep has been well-described, sleep complexity in DOC patients and its mere presence is still being debated within the research community. In healthy individuals with normal brain functioning, sleep consists of three non-REM (N1, N2 and N3) sleep stages and REM (R) sleep. Each of these stages is characterized by a typical PSG pattern and EEG graphoelements, i.e., characteristic sleep-associated brain activity micro-structures (cf. Figure 3).

Following severe brain injury, sleep-wake cycling is often highly altered (Giubilei et al., 1995; Ouellet et al., 2004; for review see Cologan et al., 2010). One important aspect that must be kept in mind is that some of the sleep abnormalities observed in critically ill patients have their origin not only in brain damage. Another important factor is also the with regard to circadian rhythmicity rather unfriendly environment of intensive care units (ICUs), where medical procedures are performed at all hours, noise levels are constantly high and ambient light of high intensity is present even during the night (Parthasarathy and Tobin, 2004; Seifman et al., 2014). Acute illness, mechanical ventilation, discomfort, pain, exposure to (sometimes constant) light and hence the loss of light as a “Zeitgeber” as well as noise or nursing activities around the clock might trigger awakenings, enhanced arousals and thus sleep fragmentation (Cologan et al., 2010). De Weer et al. (2011) were for example able to show that motor activity and arousal in DOC patients correlated significantly with changes in light irradiation suggesting that light on its own is able to transiently increase patients’ arousal levels across all DOC states. This is especially problematic when considering the negative effects of sleep deprivation on, for example, activity of the immune and endocrine systems, catabolic rate and cell division (Bryant et al., 2004; Seifman et al., 2014), which may also impair recovery from brain injury. As we will argue below, stimulation with bright light during the day could serve the re-establishment of circadian rhythms by boosting arousal during the day and promoting sleep during the night. This could hence support rehabilitation and recovery in these patients.

Fine-grained analyses of sleep using PSG could also serve the diagnostic and prognostic process. However, determining sleep and distinguishing different sleep phases in DOC patients is most challenging since they seldom show signs of sleep in EEG, EMG and EOG, which are used for sleep scoring in healthy individuals. Due to damages in specific brain areas and disruptions in neuronal pathways, characteristic EEG graphoelements may differ significantly in terms of frequency, length, or intensity, if they occur at all. Additionally, recording PSG signals of acceptable quality in DOC is extremely challenging because of artifacts caused by e.g., sweating due to dysregulations of the vegetative nervous system, spasms causing muscle artifacts, uncontrolled eye movements or electrical artifacts caused by medical equipment. Generally, these patients experience more frequent awakenings and arousals than healthy individuals and have a decreased amount of rapid eye movement (REM) as well as deep N3 NREM sleep as far as assessable (Ouellet et al., 2004). All these reasons render the classical determination of sleep stages according to the standard criteria (Iber, 2007) very challenging or even impossible.

In line with this, the literature does not draw an entirely conclusive pattern of the PSG graphoelements and their association with the patient’s state of consciousness (see Table 1). In one study, Cologan et al. (2013) observed slow oscillations (although often below 75 μV) in all, REM only in three and sleep spindles (although often below 10 Hz) in seven out of ten VS/UWS patients. Additionally, they observed sleep spindles (again, in some patients below 10 Hz), slow oscillations and REM in all MCS and MCS+ patients. Landsness et al. (2011) found slow oscillations and sleep spindles in all of six and REM in five of six MCS patients, but neither sleep spindles nor slow oscillations nor REM in any of the five VS/UWS patients examined. Finally, de Biase et al. (2014) investigated sleep in 27 VS/UWS patients and observed sleep spindles in fifteen (56%), REM sleep in four (15%) and K-complexes in 22 (81%) of them. In all five MCS patients they examined they found sleep spindles, REM and K-complexes.

**Table 1:** **EEG graphoelements and the patients’ state of consciousness**.

	Cologan et al. (2013)			Landsness et al. (2011)		de Biase et al. (2014)	
	VS/UWS	MCS	MCS+	VS/UWS	MCS	VS/UWS	MCS
**Slow-Oscillations**	10/10	7/7	3/3	0/5	6/6	—	—
**Spindles**	7/10	7/7	3/3	0/5	6/6	15/27	5/5
**REM**	3/10	7/7	3/3	0/5	5/6	4/27	5/5
**K-Complexes**	—	—	—	—	—	22/27	5/5

*Overview of results reported in the literature*.

In summary, these findings, although not being entirely conclusive, suggest that sleep in DOC patients has many facets. Generally, the results support the notion of a relationship between the presence of sleep and (partly) preserved brain functioning as well as higher levels of awareness. On the other hand, sleep does also have beneficial effects on brain plasticity and therefore supports the recovery process. Regarding sleep in LIS patients, the literature is especially scarce. Besides almost normal sleep patterns in some patients, there are also LIS patients who do exhibit hyposomnia, disorganized NREM sleep or complete REM absence (Cummings and Greenberg, 1977). This rather broad spectrum most likely arises from differences in the exact location and extent of brain lesions underlying the patient’s state. More specifically, it seems that the more widespread the pontine lesion, which is one of the possible causes of LIS, the more pronounced the sleep disturbances, especially regarding REM. Severity of sleep disturbances also increases in case of bilateral or dorsal extensions of pontine lesions, when the pontine tegmentum is involved and especially if the serotonin-releasing brainstem raphe nuclei are affected (for review see: Cologan et al., 2010).

Still, assessment of sleep and its characteristics seems to be a promising approach with regard to diagnosis and prognosis. A more systematic description of PSG patterns in DOC could furthermore benefit the establishment of DOC-specific sleep scoring criteria, which could help circumventing the limitations described above. However, even without such criteria, the mere presence of certain sleep elements and sleep stages resembling healthy sleep holds valuable information. It can allow for inferences regarding the preservation of specific brain areas and consequently brain integrity. It has, for example, been suggested that the presence of slow oscillations during sleep suggests intact functioning of certain brainstem nuclei and thalamo-cortical loops (Dang-Vu et al., 2008) and that the presence of REM may indicate integrity and preserved functioning of the brainstem pontine tegmentum (for review see: Cologan et al., 2010). Furthermore, the intensity or the amount of sleep spindles observed in the EEG of DOC patients is an indicator of intact and efficiently connected thalamo-cortical networks (for review see: Cologan et al., 2010). The authors even suggested that the shape of slow oscillations or the frequency of sleep spindles in DOC patients might reflect the preservation of the thalamo-cortical system as a whole and might thus be informative regarding the state of consciousness (Cologan et al., 2013). Also signal complexity during sleep seems to hold information about brain integrity. Valente et al. (2002) classified DOC patients’ sleep EEG patterns into five categories based on signal complexity and related these to the eventual outcome. The categories were defined as follows with higher numbers denoting higher signal complexity: (i) monophasic; (ii) cycling alternating pattern; (iii) rudimentary sleep; (iv) NREM sleep; and (v) REM sleep. Afterwards, the authors related these categories to the outcomes of 24 patients: five of the six patients who belonged to the fifth and most complex category showed good recovery; three patients from the fourth category improved, but were still severely disabled. Patients from the three least complex categories (seven patients) had an unfavorable outcome. They remained in coma, did not emerge from VS/UWS, or died.

The literature reviewed here shows that the results do not allow drawing a clear picture regarding sleep in DOC so far, neither do they allow distinguishing different DOC states taking into account the presence or absence of sleep stages and/or sleep elements. This probably results from the heterogeneity of the group of DOC (VS/UWS and MCS) patients: etiology (traumatic vs. non-traumatic), location and extent of brain lesions, time since brain injury, received medication and rehabilitation granted to a patient. Last but not least, this probably is—at least partly—also due to problems regarding the applicability of the available scoring criteria to sleep in DOC patients. However, it seems that more complex sleep patterns are related to preserved brain functioning and therefore also to a better diagnosis and prognosis, wherefore we believe it is worth investigating sleep in DOC in more detail.

### Interventions—translation of current knowledge into clinical applications

Taking into account the literature reviewed so far, we propose that bright light stimulation might be a promising tool to be utilized in DOC states. Bright light therapy could promote arousal, especially during assessment of potentially retained cognitive abilities. Furthermore, it may also help re-establish circadian rhythmicity and sleep in DOC.

It is well known that, besides synchronizing the circadian clock and regulating melatonin secretion (Thapan et al., 2001), light has a modulatory effect on arousal and activity in cerebral attention networks (Cajochen et al., 2000). On a cellular level, this regulatory mechanism involves melanopsin-expressing retinal ganglion cells (mRGCs), so-called non-image forming photoreceptors in the mammalian retina, which detect changes in environmental light irradiance. Exposure to bright light has previously been shown to promote alertness in healthy participants (Cajochen et al., 2008). Moreover, it has been found to improve concentration and working memory (Kretschmer et al., 2012) as well as general cognitive functioning (Royer et al., 2012) in elderly individuals. De Weer et al. (2011) suggested in a recent study that light therapy may also transiently promote arousal in all states of unconsciousness. In summary, we believe that bright light therapy could be a low-cost and non-invasive therapeutic approach to boost arousal and activate remaining cognitive resources in DOC patients that could, moreover, be integrated easily into clinical routine. It could, additionally, also increase the reliability of clinical assessments. For this purpose, bright light stimulation should take place right before the assessment of consciousness levels with behavioral scales or neuroscientific paradigms.

Besides the effects on cognition, in patients suffering from dementia, bright light therapy has been shown to have a positive effect on sleep (Campbell et al., 1995; Ancoli-Israel et al., 2003). This could be the result of the re-establishment of circadian rhythmicity, which is often impaired in dementia (Volicer et al., 2001), by attuning melatonin excretion. We propose that also DOC patients could benefit from bright light therapy during the day in combination with low light levels during the night. This will, according to our reasoning, benefit the restoration of circadian rhythmicity and normalize sleep, both of which have been shown to be related to a positive outcome.

## Conclusions

During the last years, an increasing amount of studies have investigated cognition in DOC patients from various perspectives and tried to find traces of consciousness. Patients who emerge from coma following severe brain injury and who do present with periods of eye-opening are considered to be in VS/UWS. By definition, they are, however, not aware of themselves and/or their environment. When patients exhibit more complex behavior such as visual pursuit or instruction-following, they are thought to be minimally conscious and to have regained some level of awareness. The challenge clinicians as well as scientists, however, face is how awareness can be detected and in a next step quantified. Despite considerable progress in this field, until now, researchers have not been able to develop a method or test battery that allows for reliable and unambiguous conclusions regarding the level of consciousness a patient retains.

It is increasingly accepted, that certain brain responses, the presence of circadian rhythmicity or of certain sleep elements as well as functional resting state activity can serve the diagnostic and prognostic evaluation process as indicators of retained brain functioning or even consciousness. For example, the absence of the earliest ERP components (i.e., somatosensory or auditory evoked potentials) has been associated with a negative outcome, whereas the presence of later components (e.g., MMN or P300) suggests preserved cognitive functioning and predicts good outcome. Active paradigms, in which patients are instructed to follow commands (e.g., count the number of presentations of a stimulus or imagine a motor action) allow for conclusions about retained higher cognitive functions (e.g., working memory, sustained and selective attention). They are, thus, useful for the detection of conscious awareness in behaviorally unresponsive patients and especially in patients at the upper end of the DOC continuum. Resting-state activation and connectivity within the DMN have been suggested to be related to two different kinds of awareness: external (regarding the environment) and internal (including reflections about oneself) awareness. It has, moreover, been shown that the EEG spectrum at rest, entropy-based measures of signal complexity as well as the combination of EEG and TMS may allow for a refined diagnosis and serve as predictors of outcome. Additionally, we propose that circadian rhythmicity and sleep in DOC should be investigated more thoroughly as they could allow differentiating between VS/UWS and MCS patients as well as inform about the integrity of certain brain areas. Last but not least, interventions aiming at re-establishing circadian rhythmicity and sleep in DOC patients such as bright light stimulation may depict new methods supporting rehabilitation.

## Conflict of interest statement

The authors declare that the research was conducted in the absence of any commercial or financial relationships that could be construed as a potential conflict of interest.
